# Contextual Modulation of Primary Visual Cortex by Temporal Predictability During Motion Extrapolation

**DOI:** 10.1002/brb3.70769

**Published:** 2025-08-22

**Authors:** Camila Silveira Agostino, Herman Hinrichs, Toemme Noesselt

**Affiliations:** ^1^ Department of Biological Psychology Otto‐von‐Guericke‐Universität Magdeburg Magdeburg Germany; ^2^ Department of Neuropsychology Otto‐von‐Guericke‐Universität Magdeburg Magdeburg Germany; ^3^ European Structural and Investment Funds‐International Graduate School (ESF‐GS) Analysis, Imaging, and Modelling of Neuronal and Inflammatory Processes (ABINEP) International Graduate School Otto‐Von‐Guericke‐Universität Magdeburg Magdeburg Germany; ^4^ Center For Behavioral Brain Sciences Otto‐von‐Guericke‐Universität Magdeburg Germany; ^5^ Department of Neurology Otto‐von‐Guericke Universität Magdeburg Magdeburg Germany; ^6^ Department of Behavioral Neurology Leibniz Institute for Neurobiology Magdeburg Germany

## Abstract

**Background:**

Predicting future events is a fundamental cognitive ability that often depends on the volatility of the environment. Previous studies on apparent motion reported that when the brain is confronted with low levels of predictability, activity in low‐level sensory areas is enhanced, including the primary visual cortex, while others suggest that the enhanced activity in this area is independent of the predictability level. Consequentially, it remains unclear how temporal predictability modulates brain responses in continuous, thus more ecologically valid, motion paradigms.

**Purpose:**

Our study investigated whether motion extrapolation in high and low predictable contexts would differently modulate fMRI responses in subject‐specific primary visual cortex during visible and partially occluded stimulation.

**Methods and Materials:**

Eighteen participants performed a modified version of the interception paradigm in visible and occluded phases, in which they observed a stimulus moving horizontally, then vertically at two different velocities, while fMRI data was acquired. They judged when and where the stimulus would reach a given point of contact. In a high predictable context, the velocity was identical during horizontal and vertical (occluded) movement; whereas, in a low predictable context, the velocity could change during the vertical trajectory, introducing a temporal incongruence to the task with a predictive role of velocity on the trajectory estimation.

**Results:**

Univariate results indicated that on average both low and high predictable contexts similarly modulated activity in primary visual areas. On the other hand, trial‐history analysis showed that a change in trial type (constant velocity after change in velocity and vice versa) increased BOLD responses in V1.

**Conclusion:**

This pattern of results suggests that motion extrapolation can modulate activity in the primary visual cortex regardless of average predictability but is influenced by recent trial history. These results were further supported by multivariate pattern analysis, which revealed different patterns when comparing congruent and incongruent trials in the context of lower predictability.

## Graphical abstract

This study explored how motion extrapolation in high‐ and low‐predictable contexts modulates fMRI responses in the primary visual cortex. While predictability had a similar average effect, trial‐history analysis revealed increased BOLD responses following trial type changes. Multivariate analysis further highlighted differences in incongruent trials under lower predictability.

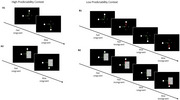

## 1 Introduction

Completing information partially missing from the environment—e.g., due to occlusion—is among the daily challenges that the cognitive system has to deal with, and for that, reliable interpolation or predictive mechanisms are crucial. Prediction, as used here, can be understood as the ability of the brain to estimate future input based on (recent) past information (Alink et al. 2010). Earlier studies developed theoretical models to explain how our brain works in a predictive fashion (Mumford 1992; Rao and Ballard 1999; Friston 2003). One model that gained attention was the predictive coding model from Rao and Ballard (1999). In brief, the model assumes that different levels of a hierarchical model network make predictions and send them to lower levels via feedback connections, while higher levels receive back the information about the error between the prediction and the actual response via feedforward connections. This error is also used by the system to make corrections on the estimation of the input signal. This process would explain how predictions are coded in visual regions: lower visual areas learn statistical regularities from the environment and send forward the unpredicted aspects of the received input. Besides Rao and Ballard, others also attempted to mathematically explain how different systems work, by varying how the model is applied to the data and how the error is minimized (for review, see Spratling 2017; Aitchison and Lengyel 2017). Predictive coding models have been used to test the mechanisms underlying temporal and spatial predictions in different domains, such as auditory (Baess et al. 2009; Heilbron and Chait 2018, for review), motor (Shipp, Adams, and Friston 2013), multisensory integration (Krala et al. 2019), and the visual domain.

In the visual domain, Alink et al. (2010) directly tested the predictive coding model by investigating whether a highly predictable moving visual stimulus decreases activity in the primary visual area and whether the stimulus predictability also influences regions that send feedback information to V1, such as hMT/V5+ (Vetter et al. 2015). The authors used an apparent motion task and a random dot motion paradigm to test how unpredictable temporal and spatial information, respectively, enhanced fMRI responses in V1. For apparent motion, highly predictable stimulus onsets indeed reduced activity in V1, compared to unexpected delayed onsets. For random dot motion, they also observed a decrease in response in V1, as well as in hMT/V5+, when the random dot motion direction was predictable. These findings supported the predictive coding model by showing that temporally and spatially predictable visual stimulations reduce activity in V1, while unpredictable visual stimulation enhances activity in this region. Later, Schellekens et al. (2016) extended these findings to V2 and V3 by testing how predictable and unpredictable contrast changes can modulate neural response in low‐level visual regions, using random‐dot motion at specific locations. Results indicated that when new dots entered the visual field by being presented in new locations, higher responses were registered in V1, V2, and V3 compared to dots that were already displayed in these areas. A recent EEG study, which investigated the representation of the real‐time position of random moving dots, reported that such representations were updated in real time as the dots moved (Johnson et al. 2023), suggesting that V1 is able to update position information continuously. These findings are also in line with similar studies in humans (Ekman et al. 2017), which found anticipatory V1 responses for upcoming predicted events.

Using more complex stimuli Fischer et al. (2013) investigated how temporal predictability affects neural processing in low‐level visual areas during the categorization of predictable and unpredictable moving faces when primed by an auditory alerting signal. One group of the participants was asked to judge whether the presented moving face was male or female, while the other group judged the direction of the stimuli. In half of the trials, the alerting cue was presented before the visual stimulus. Results indicated that participants’ behavioral performance was higher when the visual stimulus onset was predictable and when the alerting signal was coherent with it. However, a negative correlation was found between activity in V1 and the alerting signal, i.e., the larger the effect of the alerting signal on behavioral performance, the stronger the reduction in the primary visual cortex. These findings suggest that the increase of temporal predictability reduced the BOLD signal in V1, corroborating previous studies and supporting the importance of temporal information in the prediction of future events.

Previously, we found that predicting partially occluded motion trajectories in a highly predictable environment lead to enhanced fMRI responses in V1 and—most importantly—decodable informative patterns of activity (Agostino et al. 2023). However, it remained unclear how a manipulation of volatility would affect this pattern of results.

In this study, we aim at probing the influence on temporal stimulus predictability in the context of motion extrapolation with a continuous and complex trajectory with a 90° turning point. To this end, we investigated whether dynamically occluded stimulation presented in high predictable (HP) and low predictable (LP) contexts would differently modulate fMRI signals in V1 by manipulating temporal information. We hypothesized that, according to the predictive coding theory, the signal in this region should be smaller during stimulus presentation in the HP context due to a higher level of volatility compared to the LP context. To test this hypothesis, we used an adapted version of the interruption paradigm (IP—Battaglini and Ghiani 2021), monitored brain activity using fMRI, and acquired retinotopic maps in order to identify subject‐specific regions of interest. In addition to univariate analysis, multivariate pattern analyses were carried out in right upper and lower V1 (and right hMT/V5+, see Supplementary Material), as the stimulation was presented only in the left hemisphere, in order to investigate changes in the representational pattern of activity in these regions (see Agostino et al. 2023 for a similar approach).

## 2 Materials and Methods

### 2.1 Participants

Eighteen participants (mean age 25.5, ± 4.23, eight women) with normal or corrected‐to‐normal vision, no history of psychiatric or neurological disorders and no regular intake of medication known to interact with the central nervous system were recruited from the student community of Otto‐von‐Guericke Universität Magdeburg and gave informed consent to participate in the study according to the local ethics. In a two‐day experiment, participants were exposed to eight tasks, four each day, while their brain activity was monitored by fMRI: two training phases (visible stimulation) and two tests phases (occluded stimulation). On a third day participants returned for a retinotopic mapping measurement. Volunteers were rewarded with 10 euros/hour or experiment credits. Two participants were excluded due to incomplete data acquisition.

### 2.2 Task

**FIGURE 1:**
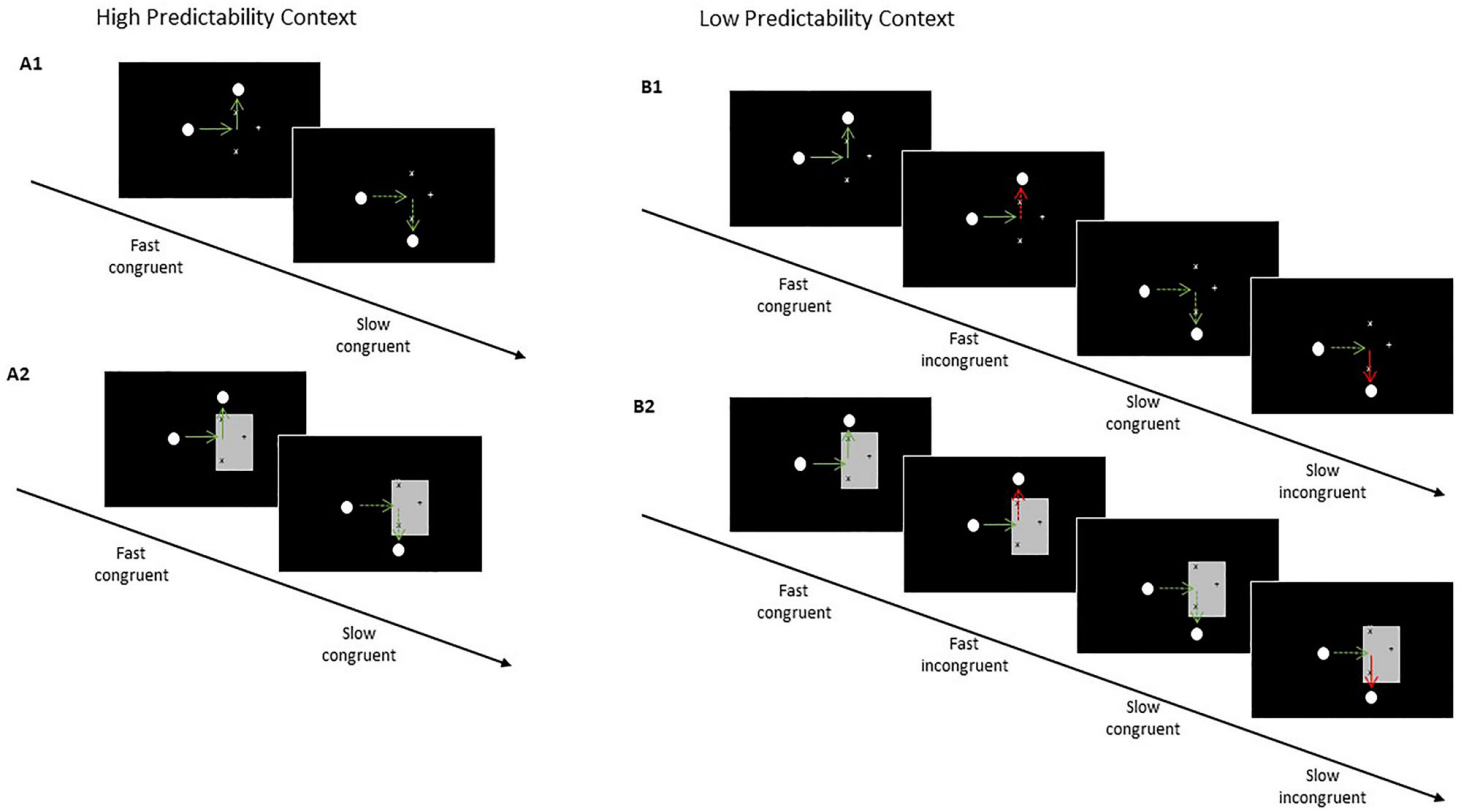
Experimental paradigms: **(A1)** High predictability (HP) context of the visible phase: The full and dotted green lines (not presented during the experiment) represent fast and slow velocities, respectively. Participants saw the white dot moving from the left side of the screen to the center and turning 90° upwards or downwards. The vertical and horizontal trajectories had the same velocity (upwards‐fast, downwards‐slow, or upwards‐slow, downwards‐fast). They were instructed to press the right or left button the moment that the dot reached the constantly present upper or lower “x” marks (according to the instructions), **(A2)** High predictability context of the occluded phase: In this phase, a grey rectangle was introduced to the task scenario, occluding the vertical trajectories. Participants observed the same velocity‐direction pairings learned during the visible phase and were asked to press the button when the dot reached the “x” mark and to refrain from pressing the button once the dot had reappeared from behind the rectangle, **(B1)** Low predictability (LP) context of the visible phase: this phase contained the same stimuli as the HP context, but in 30% of the trials, the velocity‐direction pairing was incongruent (represented by the red arrows), i.e., the dot changed velocity at the upward/downward turning point. The participants received exactly the same instructions as in the LP context task and learned about the incongruency factor throughout the trials, and **(B2)** LP context in the occluded phase: During this phase, the participants performed the exact same task as in the visible phase, with the difference that the vertical trajectory was occluded by a grey rectangle again.

*High Predictable Context—Visible Phase (training)*: On a black screen, a white dot moved continuously horizontally (200 px) from the left side to the center, then vertically upwards (200 px) or downwards (200 px) until it crossed an “X” mark (+120 px) (see Figure 1A). The vertical direction would be determined by the velocity of the horizontal movement, which could be 16.6°/s (fast, 0.250 s; 0.150 s after crossing the “X” mark) or 14.4°/s (slow, 0.450 s; 0.266 s after crossing the “X” mark). [Note that the velocities (or time displacement) used until the stimulus reached the “X” mark were the intervals used for modeling and comparison with participants’ responses]. Hence, participants could easily learn the dependency of trajectory change on velocity since velocity was constant during horizontal and vertical trajectories. The participants were asked to keep their eyes on the fixation cross while attending to the sequence of movements and to indicate where and when the dot would end. We asked them explicitly to avoid pressing after the stimulus crossed the marks. Subjects responded by pressing the left button with their index finger or the right button with their middle finger in case the dot reached the upper “X” mark or lower one, respectively. Thirty trials of each condition were presented in four runs, i.e., 240 trials total (two runs with the configuration: up‐fast/down‐slow and two runs with up‐slow/down‐fast). The intertrial interval was chosen from a Poisson distribution with values between 2 and 6 s, and each run lasted around 8 min.

*High Predictable Context—Occluded Phase (test)*: Our adaptation of the IP task was very similar to the visible task described above, with the difference that a grey rectangle, occluding the vertical trajectory, was presented during the whole trial. Specifically, participants received the following instructions: “In this phase, you will be presented with a white dot moving from the left side to the center of the screen. At the same time, there will be a grey rectangle on the screen occluding the following movements of the dot. On this rectangle, there will be two black “X” marks on the trajectories of the moving dot. Please, attend to the movement while you keep your eyes on the fixation cross. Your task is to press the button when the dot reaches the “X” marks. Do not follow the dot with your eyes, and do not answer after its reappearance! Use the left button for UPWARD movements and use the right button for DOWNWARD movement.” Participants extracted the temporal information from the horizontal trajectory in order to make the judgment about the stimulus destination and time of contact (Figure 1B). Fifty trials of each condition were presented in four runs, resulting in 400 trials (same configuration as above). The intertrial interval was chosen from a Poisson distribution with values between 2 and 6 s, and each run lasted around 13 min (Hinrichs et al. 2000).

*Low Predictable Context—Visible Phase (training)*: In this session, we manipulated the task predictability by introducing different probabilities of the temporal information. To this end, the paradigm in this phase remained the same as described above with the following exceptions: if the stimulus moved slowly along the horizontal trajectory, it could change speed in 30% of the trials and move faster along the vertical trajectory, or vice versa. Seventy‐two out of 240 trials were presented with the incongruent displacement time, and the number of trials was counterbalanced across conditions. Note that the overall duration of speeding up incongruent trials and slowing down incongruent trials in the vertical trajectory was kept constant (0.716 s), until the point of contact.

*Low Predictable Context—Occluded Phase (test)*: The task for this phase remained the same as described in the test phase of the high predictable context, with the changes in velocity changes already reported above in the last section (visible phase of the low predictable context). The challenge in this phase was to estimate the displacement time without having the visual information of the vertical trajectory. The stimulus reappearance from behind the occluder served as feedback, but participants were instructed to provide their responses prior to reappearance, and responses after reappearance were counted as misses. One hundred and twenty out of 400 trials contained the incongruent displacement time, and the number of trials was counterbalanced across conditions.

### 2.3 fMRI Data Acquisition

The scanning sessions were conducted on a 3 Tesla Siemens PRISMA MR system (Siemens, Erlangen, Germany), using a 64‐channel head coil. The data of participants were acquired in 16 functional runs divided into two sessions, totaling 1904 volumes for the training phases and 3144 volumes for the test phases for each subject^1^. Blood oxygenation level‐dependent (BOLD) signals were acquired using a multi‐band accelerated T2*‐weighted echo‐planar imaging (EPI) sequence (multi‐band acceleration factor 2, repetition time (TR) = 2000 ms, echo time (TE) = 30 ms, flip angle = 80°, field of view (FoV) = 220 mm, voxel size = 2.2 × 2.2 × 2.2 mm, no gap). Volumes were acquired in interleaved order. Identical slice selection on both days was achieved using Head Scout Localizer, whose calculation is based on Autoalign (Siemens, Erlangen).

A high‐resolution three‐dimensional T1‐weighted anatomical map (TR  =  2500 ms, TE  =  2.82 ms, FoV  =  256 mm, flip angle  =  7°, voxel size  =  1 × 1 × 1 mm, 192 slices, parallel imaging with a GRAPPA factor of 2, and 5:18 min scan duration) covering the whole brain was obtained using a magnetization‐prepared rapid acquisition gradient echo (MPRAGE) sequence. This scan was used as an anatomical reference for the EPI data during the registration procedure.

### 2.4 Retinotopic Mapping

The procedure used for measuring the retinotopic maps was similar to the one used by Warnking et al. (2002) and Bordier et al. (2015). Eccentricity was mapped using a checkerboard ring, which slowly contracted or expanded from the fixation dot while presented on a grey background. The speed of the expansion and the contraction varied linearly with the eccentricity, so that the activation wave kept travelling at an approximately constant speed (Bordier et al. 2015). The ring reached a maximum diameter eccentricity of 6.6° and a minimum of 0.2°. When the maximum (expansion) or the minimum (contraction) was reached, a new ring would start from the origin. Polarity was mapped using one checkerboard wedge (10°) slowly rotating at a constant speed. Specific parameter calculations were similar to the ones described by Warnking et al. (2002). The checkerboard stimulation flickered at a frequency of 8 Hz, in ten cycles of 36 s each. The aspect ratio of the checkerboards was kept constant (1.09) by scaling the height linearly with the eccentricity. In order to account for the effects of the hemodynamic delay, the wedges were presented clockwise and counterclockwise, and the rings were presented asexpanding annuli and contracting annuli (Warnking et al. 2002). In total, eight functional runs were acquired, two for each modality and direction, and each run lasted approximately 6 min. Data acquisition was done using a multi‐band accelerated T2×‐weighted EPI sequence (multi‐band acceleration factor 2, TR = 2000 ms, TE = 30 ms, flip angle = 90°, FoV = 128 mm, voxel size = 2.2 × 2.2 × 2.2 mm, no gap). We acquired 180 volumes in interleaved order for each run.

## 3 Statistical Analysis

### 3.1 Behavior

Participants’ performance was assessed through the averaged correct responses (accuracy), response time, and response time error (difference between individual's response time and the presented stimulus duration). Trials with RTs exceeding 0.65 s were excluded (i.e., < mean plus 3 sd). These trials may have been contaminated by the information from the reappearing stimulus and had a mean RT = 0.864, (SE ± 0.027). On average 7.14 (SD ± 9.83) of trials were excluded per subject.

The three measurements were calculated for training and test phases and used as input for three different repeated measures (RM) ANOVAs: (1) For the HP context, we used a 2×2 within‐subject design (direction: up‐down vs. velocity: fast‐slow); (2) for the LP context, we used a 2×2×2 within‐subject design (direction: up‐down vs. velocity: fast‐slow vs. congruency: congruent‐incongruent); (3) for the comparison of HP versus LP contexts, we carried out a 2×2×2 within‐subject design as well (direction: up‐down vs. velocity: fast‐slow vs. predictability: high vs. low predictable) using the congruent trials only, as no incongruent trials were present in the HP context. Note that, when presenting the LP context results, we refer to the trials as incongruent fast whenever the stimulus travelled the horizontal trajectory fast and was *slowed down* during the vertical trajectory in the visible or occlusion phases, and as incongruent slow when the stimulus travelled the horizontal trajectory slow and was *sped up* during the vertical trajectory in both phases, i.e., we use the horizontal speed for our terminology. Additionally, for each analysis, we included task order as a between‐subject factor; however, in no statistical analysis was any significant effect observed (all *p*’s > 0.213). All analyses were calculated using JASP (v.0.15.0—https://jasp‐stats.org/). JASP was also used to compute post hoc tests and effect sizes (partial ƞ^2^).

### 3.2 Retinotopy

We performed a three‐dimensional reconstruction of the cortical sheet based on the structural image of each of the 16 subjects using the recon‐all function from FreeSurfer (v.6—https://surfer.nmr.mgh.harvard.edu/). Retinotopic maps along the polar and eccentricity dimensions were calculated for each of the cortical surfaces using the “selxavg3‐sess” function from FreeSurfer. Lower and upper primary visual areas were delineated manually on the flattened cortical sheets based on the boundaries of phase reversals within the polar angle and eccentricity maps (Abdollahi et al. 2014). Delineation of borders was created based on Georgieva et al. (2009) and Kolster et al. (2010). The regions were later used to identify the local maxima during the visible phase in the HP context separately for lower and upper V1. These local maxima were thus used to independently localize the region of interest within the functional region for the occluded phase. Probabilistic maps of MT as provided by FreeSurfer parcellation were used for the identification of motion‐sensitive regions.

### 3.3 fMRI Preprocessing

All data (except retinotopic data) were analyzed using SPM12 (www.fil.ion.ucl.ac.uk/spm, Wellcome Trust Centre for Neuroimaging, London, UK). The first five volumes of each run were discarded to allow for steady‐state magnetization. We performed slice‐timing correction and realignment (registered to the mean image) of all remaining functional volumes. Head motion parameters were later used as nuisance regressors in the general linear model (GLM). Finally, the structural image was coregistered (estimated and resliced) to the first functional image of the first run. Resliced images were smoothed with a Gaussian kernel of 6 mm.

### 3.4 fMRI Data Modeling

Data of individual contexts (HP and LP) were modeled with a general linear model (GLM, Friston et al. 1995), which included the run‐wise condition parameters, derivatives, and six motion regressors as nuisance covariates. In particular, regressors of each condition (up‐fast, down‐slow, up‐slow, down‐fast, up‐slow, down‐fast, up‐fast, down‐slow, and the respective incongruent conditions of LP contexts) were modeled with the canonical hemodynamic response function (HRF), using the onset of the initial stimulus trajectory of each trial. Temporal and dispersion derivatives of each regressor were added to the model in order to account for variability in the onset response and shape (Friston et al. 1998). Estimated beta weights of the HRF of each participant were extracted using MarsBar 0.44 (Brett et al. 2002) from subject‐specific lower and upper V1 masks (see below for details of retinotopic analysis). Importantly, voxels containing response signals related to the reappearance of the stimulus were excluded from the data. We performed three rmANOVAs: (1) for HP context, we used a 2×2×2 (direction, velocity, V1 Quadrant); (2) for LP context, we used a 2×2×2×2 (direction, velocity, V1 Quadrant, and congruency) and for combined congruent HP and LP, a 2×2×2×2 (direction, velocity, V1 Quadrant, and predictability). Task order was included in the analysis as a between‐subject effect, but no significant effects for this factor were observed (all *p*’s > 0.078). A full rmANOVA (direction, velocity, predictability, V1 quadrant, and task order) was run for comparisons in which all tasks were included, however, no difference between tasks were observed. Results are presented in the supplementary material, Table 1. For the multivariate pattern analysis (MVPA), we modeled single‐trial GLMs using a least square separate (LSS) approach, (Mumford, 2012—script adapted from https://github.com/ritcheym/fmri_misc/blob/master/generate_spm_singletrial.m).

**TABLE 1:** Reaction time results. Post‐hoc analyses of the main results of the 2×2×2 repeated measures ANOVA for visible and occlusion phases as described above. (See Supplementary Material for further results).

Behavior: Reaction time (s)							
Phase	Predictability	Congruency	Direction	Velocity	Mean Difference	Post‐hoc	p_bonf_
**Visible**	**High**	Congruent	*	Fast > Slow	−0.106 (± 0.009)	*t*=‐11.982	< 0.001
			Upward > Downward	*	−0.032 (± 0.008)	*t*=‐3.842	0.002
	**Low**	Congruent > Incongruent	Upward	Fast	−0.106 (± 0.014)	*t*=‐7.539	< 0.001
			Downward		−0.073 (± 0.014)	*t*=‐5.168	< 0.001
			Upward	Slow	0.061 (± 0.014)	*t*=4.313	0.002
	**High > Low**		Downward		0.073 (± 0.011)	*t*=6.457	< 0.001
**Occlusion**	**High**	Congruent	*	Fast > Slow	−0.112 (± 0.013)	*t*=‐8.886	< 0.001
			Upward > Downward	*	−0.023 (± 0.006)	*t*=‐3.889	0.002
	**Low**	—	—	—	—	—	—
	**High > Low**		*	Slow	0.079 (± 0.013)	*t*=6.015	< 0.001

* Results were average over the respective level.

### 3.5 Multivariate Pattern Analysis

A series of trial‐wise multivariate pattern analyses was performed on beta values from GLMs of low and high predictable context for both visible and occluded phases, using CoSMoMVPA (Oosterhof et al. 2016). To this end, trial‐wise GLMs were carried out, and for the MVPA, the trials were calculated using the LSS approach (Mumford et al. 2012). Trial‐wise MVPA was chosen here due to the low number of runs for each task. Two runs are not enough to make valid train and test partitions, as we would have only one in each part. For these cases, trial‐wise analyses are recommended as the number of trials allow enough data in each partition (Mumford et al. 2012). For all analyses, we used the searchlight method with a 4.4 mm sphere, a linear discriminant analysis (LDA) classifier, and leave‐25%‐trials‐out (Etzel and Braver 2013). All partitions were balanced and repeated four times. All analyses were carried out at the single‐subject level, as the searchlight analyses were performed inside each individual mask.

We applied a cutoff at the accuracy of 0.5 (chance level) to filter out all the voxels that contained below‐chance accuracies and included in the analysis only the values that belonged to the highest 5% of the distribution (Agostino et al. 2023). This additional thresholding was done in order to obtain only the most informative voxels of the decoding. This procedure was applied to all the classification analyses described below. Permutations were carried out at the subject level with 1000 iterations, which contained randomized data labels per run, keeping the same original dataset. In order to have the spatial comparison, the same searchlight spheres included in the 5% highest accuracy sample were obtained for all 1000 samples of each individual participant. These sampled values were averaged across spheres for the original and permuted dataset permutations. For group‐level analysis, analyses were done based on Etzel (2017) approach. The null distribution carried the average across participants for each of the 1000 permutations plus the true‐labeled group‐level average (1001 group‐level accuracies). The permutation *p*‐value was computed by taking the sum of the permuted accuracies higher or equal to the true‐labeled accuracy and dividing by the number of iterations plus 1. Each analysis described below served a different purpose, such as decoding spatial, temporal information from both contexts and congruency information from low predictable contexts.

*Classifying direction in HP and LP context*: Here, we carried out two classification analyses in which the classifier was trained in the visible phase and tested in the occluded phase using data of each context independently. We attempted to decode spatial visible information—upward versus downward motion trajectory—from the occluded phase and compared accuracies from both analyses. We expected accuracies from LP context classification analysis to be significantly lower than HP contexts due to the presence of incongruent trials, i.e., unpredictability.

*Classifying velocity in HP and LP context*: As the classification analyses above show, we trained the classifier on the data of the visible phase and tested on data of the occluded phase within contexts. In this case, we attempted to decode temporal visible information—fast versus slow—from occluded data and compared accuracies from the different contexts. Here, we also expected decoding accuracies from LP context to be smaller than accuracies from HP context, also due to the presence of incongruent trials.

*Manipulation checks*: To verify whether the classifier was really decoding relevant information, classification analyses were performed on the visible and occluded data separately for both contexts. For HP contexts, direction and velocity were classified from both phases, as well as for LP contexts. Additionally, for the latter, congruent versus incongruent information was classified. Here, we expected that decoding accuracies from visible phases would be higher than occluded phases, as the longer exposure of the stimulus during the visible phase may allow a more robust representation of the information, whereas during occlusion, participants are expected to mentally represent the trajectory thus, less bottom‐up input would drive the response.

We further ran pairwise Student's *T*‐tests to compare HP and LP contexts in all conditions in lower and upper V1 quadrants to investigate whether conditions in LP context would encode more or less information, hence higher or lower accuracy values, compared to HP context.

## 4 Results

### 4.1 Behavior: Temporal Estimation

#### 4.1.1 Visible Phase

**FIGURE 2:**
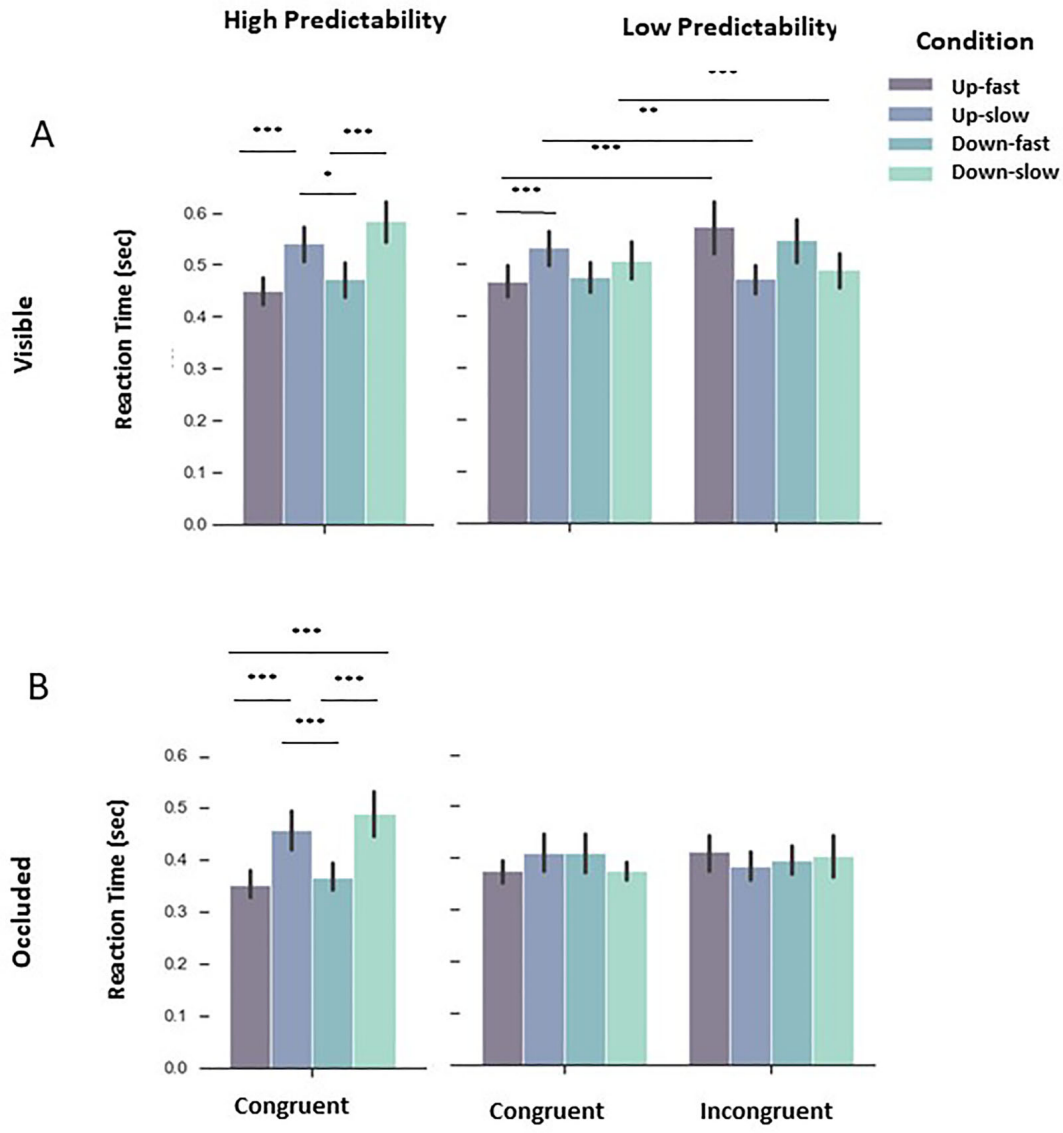
Group mean reaction times. Dark purple bars depict results of fast motion along the upward trajectory, blue bars depict results of slow motion along the upward trajectory, dark green bar depict fast motion along the downward trajectory, and light green bars depict slow motion along the downward trajectory. **(A)** Reaction time results of the visible period in HP (left) and LP contexts (right). We observed differences between fast and slow conditions along the up‐ and downward trajectories, indicating that participants were estimating the time‐to‐contact correctly (answering faster when the stimulus was fast and answering slower when the stimulus was slow). This pattern can be clearly observed during HP and LP contexts and **(B)** Results of the occluded period in HP (left) and LP contexts (right). Reaction times in the occluded HP context mirror those of the visible HP context, whereas this similarity was not found for the occluded LP context (B, right side).

*High predictable context*: Statistical analysis revealed main effects of velocity (F (1, 14) = 143.573, *p* < 0.001, ŋ_p_ = 0.911 (see Table 1 for all post‐hoc analyses), indicating that participants answered slower to slow motion, as expected, and also to the downward direction. Figure 2A shows averaged reaction time for all conditions during visible phase, for completeness.

*Low predictable context*: Figure 2A (LP context—Congruent) shows a similar RT pattern for the congruent LP context compared to the HP context. During incongruent trials the pattern apparently reversed, with stimulus slowing down in the vertical trajectory, showing even longer RTs than the slow trials in the congruent condition plus an effect of direction (Figure 2A—LP context—Incongruent). Accordingly, the statistical analysis revealed a triple interaction between direction, velocity and congruency, which indicated slower response time during incongruent in both directions, and for congruent slow in upward direction (F (1, 14) = 5.882, *p* = 0.029, ŋ_p_^2^ = 0.296; see Table 1 for post‐hoc analyses and supplementary information for further results).

*High × Low predictability*: While the pattern of results appears to be qualitatively similar for HP and LP congruent trials, the reaction times in the LP context appear to be compressed, with slow stimulus being faster and fast stimulus being slower in the vertical trajectory. Accordingly, we observed a triple interaction between direction, velocity and predictability (F (1, 14) = 5.340, *p* = 0.037, ŋ_p_^2^ = 0.276, see Supplementary Material for further results).

#### 4.1.2 Occluded Phase

*High predictable Context*: RT Results indicated main effects of velocity (F (1, 14) = 78.964, *p* < 0.001, ŋ_p_^2^ = 0.849 (see Table 1 for post‐hoc analyses)), as well as of direction (F (1, 14) = 15.200, *p* = 0.002, ŋ_p_^2^ = 0.521), pointing to higher response time to slow motion and downwards direction, similar to the pattern observed during the HP visible phase. Figure 2B shows averaged reaction time for all direction‐velocity paired conditions during the occluded phase.

*Low predictable context and High × Low predictability*: Our statistical analysis did not yield any significant results for reaction time during low predictable context, suggesting that participants used an average of the slow and fast conditions. A similar tendency was already observed during the LP visual phase with the RT compression.

Accordingly, for the direct comparison of response times in LP and HP contexts, we found an interaction for velocity and predictability (F (1, 14) = 83.224, *p* < 0.001, ŋ_p_^2^ = 0.856), suggesting higher response times for estimation for slow velocity during HP context, but lower response times for fast velocity in the HP context. This suggests that participants may have chosen a different strategy, as response times for the congruent trials showed a central tendency (see Supplementary Material for further details also of the temporal estimation error and spatial estimation, which were always close to the ceiling). In addition, it is worthy of mentioning that we also performed statistical analysis, including visible and phase analysis. This analysis revealed complex interactions between predictability, visibility (visible vs. occluded), and direction (F (1, 14) = 6.708, *p* = 0.021, ŋ_p_^2^ = 0.324; see Supplementary Material for further results), and predictability, visibility, and velocity (F (1, 14) = 23.186, *p* < 0.001, ŋ_p_^2^ = 0.624).

### 4.2 Univariate fMRI‐results

#### 4.2.1 Visible Phase

**FIGURE 3:**
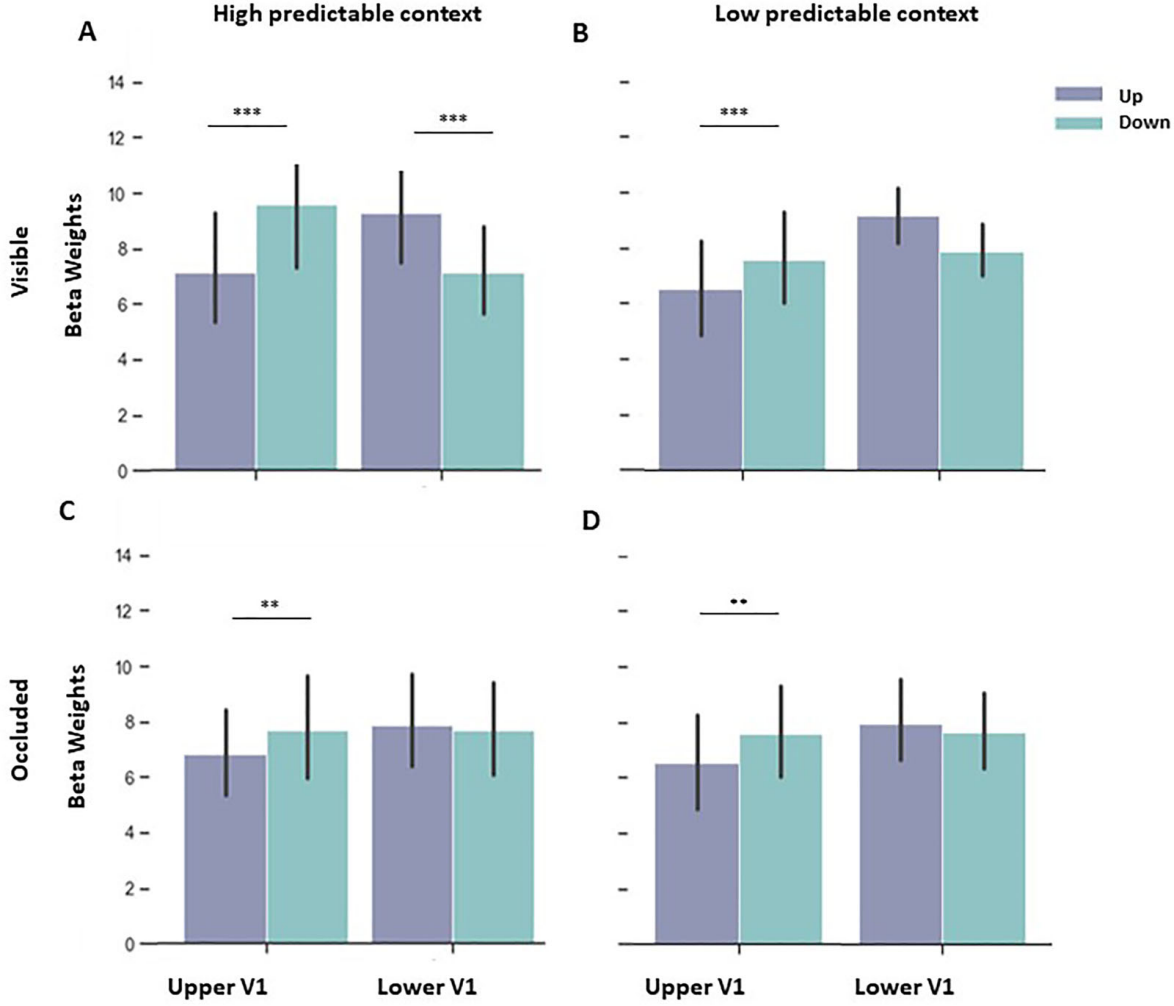
Effect of direction on V1‐quadrant‐specific BOLD‐responses. Beta weights (proportional to % signal change) of HP and LP contexts during (A) visible and (B) occluded phases, indicating an interaction between direction and V1 Quadrant. Purple bars represent upward trajectory, and green bars represent downward trajectory. **(A)** During the visible phase in the HP context, as expected, we observed that the downward trajectory enhanced responses in upper V1 compared to the upward trajectory, whereas upward trajectory elicited higher responses in lower V1 compared to the downward trajectory and **(B)** During the visible phase in the LP context, we observed the same pattern as in the HP context; however, significant results were seen only in upper V1, while results in lower V1 were just marginally significant.

**FIGURE 4:**
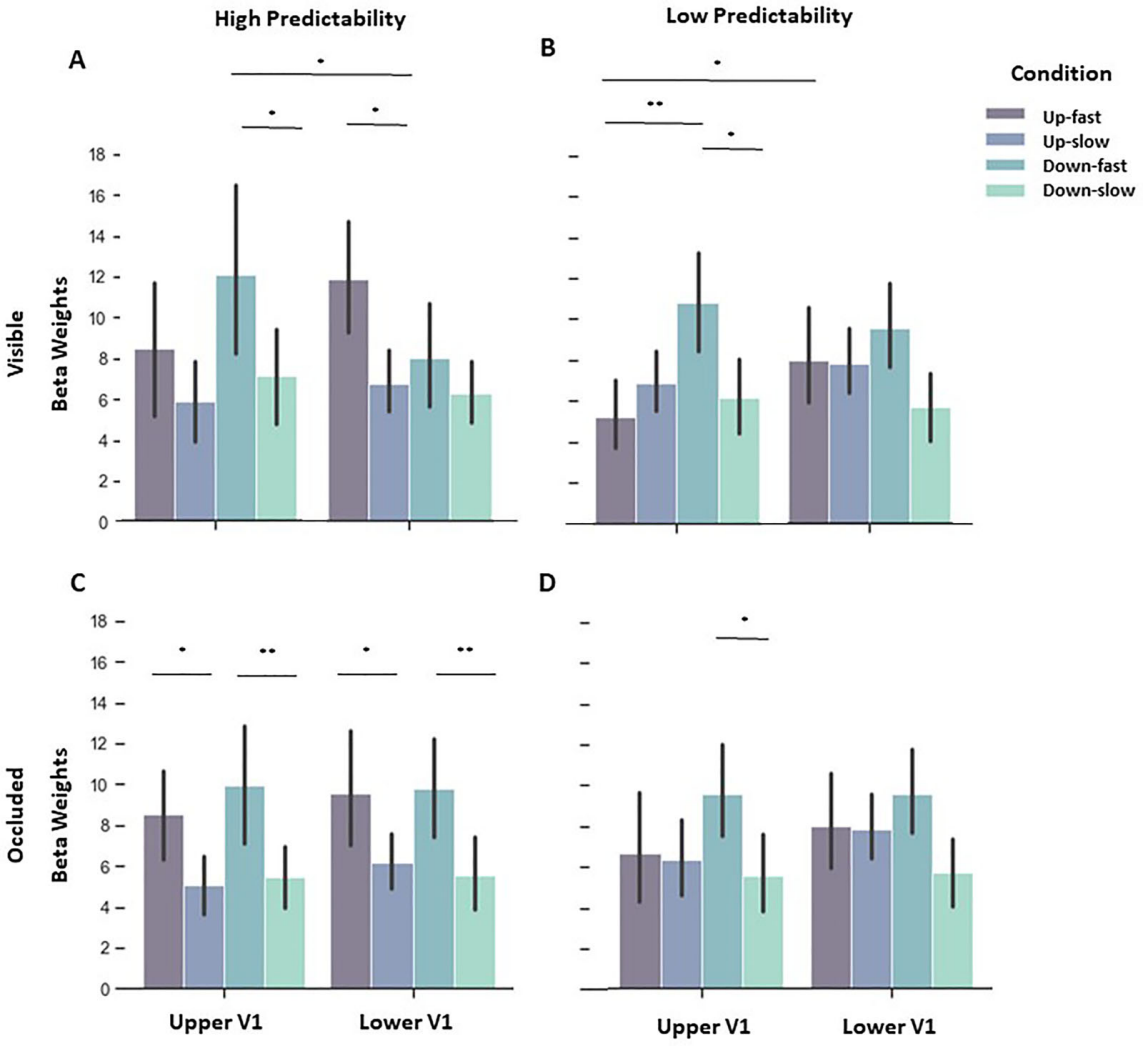
Beta weights (proportional to % signal change) of HP and LP contexts during the visible and occluded phase, indicating an interaction between direction, velocity and V1 Quadrant. Purple bars represent upward trajectories and green bars represent downward trajectories. **(A)** During the visible phase in HP context, we observed higher responses for fast compared to slow motion along downward trajectories in upper V1, which were also higher compared to fast downward in lower V1. In lower V1, responses during fast motion were higher than slow motion along upward trajectories, **(B)** During visible phase in LP context, fast motion compared to slow motion elicited higher responses for downward trajectories in upper V1, which were also higher compared to fast motion in upward direction. However, fast upward motion was higher in lower V1 compared to upper V1, **(C)** During the occlusion phase in HP context, differences were observed within the same quadrant only. Fast motion in upward, as well as in downward trajectory elicited higher responses compared to slow motion for the respective trajectories, and **(D)** In LP context, during occlusion phase, fast motion compared to slow motion in downward direction enhanced responses in upper V1.

*High predictable context*: Enhanced fMRI signals along the upward trajectory were found in lower V1 and vice versa, in accord with established theories of visual processing. Accordingly, a statistical analysis of beta weights during visible stimulation presented in the HP context indicated an interaction between direction and V1 Quadrant (Figure 3A), which suggests that the vertical stimulations were salient enough to elicit robust responses in the opposite quadrant (F (1, 14) = 76.090, *p* < 0.001, ŋ_p_^2^ = 0.845; upward vs. downward in the upper V1 (see Table 2 for post‐hoc analyses)). Moreover, a main effect of velocity was found (F (1, 14) = 35.717, *p* < 0.001, ŋ_p_^2^ = 0.718), as well as a triple interaction between direction, velocity, and V1 quadrant (Figure 4A), mainly pointing to higher fMRI responses during fast downward motion in upper V1 (F (1, 14) = 6.761, *p* = 0.021, ŋ_p_^2^ = 0.326). No effect for task order (i.e., participant starts with HP or LP context) was observed for any of the conditions.

**TABLE 2:** fMRI results of univariate analyses. Post‐hoc analyses of the main results of the 2×2×2×2 repeated measures ANOVA for visible and occlusion phases as described above. See Supplementary Material for further results.

fMRI: Univariate								
Phase	Predictability	ROI	Congruency	Direction	Velocity	Mean Difference	Post‐hoc	p_bonf_
**Visible**	**High**	*	Congruent	*	Fast > Slow	3.690 (± 0.617)	*t*=5.976	< 0.001
		Lower V1		Upward > Downward	*	2.230 (±.511)	*t*=4.364	< 0.001
				Upward	Fast > Slow	5.252 (± 1.442)	*t*=3.642	0.28
		Upper V1		Upward > Downward	*	−2.485 (± 0.511)	*t*=‐4.865	< 0.001
				Downward	Fast > Slow	5.019 (± 1.442)	*t*=3.480	0.043
		Upper V1 > Lower V1			Fast	4.200 (± 1.155)	*t*=3.636	0.031
	**Low**	*	Congruent	*	Fast > Slow	4.240 (±.374)	*t*=11.329	< 0.001
			Congruent > Incongruent		Fast	3.319 (±.455)	*t*=7.286	< 0.001
					Slow	−1.706 (±.455)	*t*=‐3.746	0.006
			*	Downward	Fast > Slow	4.212 (±1.180)	*t*=3.570	0.016
		Lower V1		Upward > Downward	*	1.304 (±.472)	*t*=2.764	0.063
		Upper V1				−2.630 (±.472)	*t*=‐5.573	< 0.001
					Fast	−5.799 (±1.284)	*t*=‐4.517	0.005
				Downward	Fast > Slow	4.766 (±1.237)	*t*=3.852	0.031
	**High > Low**	*	Congruent	*	Fast > Slow	3.965 (±.446)	*t*=8.885	< 0.001
		Lower V1		Upward > Downward	*	1.515 (±.456)	*t*=3.320	0.016
				Upward	Fast > Slow	4.075 (±1.137)	*t*=3.585	0.004
		Upper V1		Upward > Downward	*	−2.777 (±.456)	*t*=‐6.087	< 0.001
					Fast	−4.965 (±1.118)	*t*=‐4.441	0.005
				Downward	Fast > Slow	6.158 (±1.137)	*t*=5.418	< 0.001
**Occlusion**	**High**	*	Congruent	*	Fast > Slow	4.013 (±.613)	*t*=6.547	< 0.001
		Lower V1		Upward		3.623 (±1.054)	*t*=3.439	0.044
				Downward		4.230 (±1.054)	*t*=4.015	0.009
		Upper V1		Upward > Downward	*	−0.945 (±.285)	*t*=‐3.315	0.16
				Upward	Fast > Slow	3.667 (± 1.054)	*t*=3.480	0.039
				Downward		4.531 (± 1.054)	*t*=4.301	0.004
	**Low**	*	Congruent > Incongruent	Downward	*	1.462 (±.349)	*t*=4.192	0.003
				*	Fast	2.767 (± 0.461)	*t*=6.004	< 0.001
			Congruent		Fast > Slow	3.953 (± 0.602)	*t*=6.561	< 0.001
			*	Downward		4.119 (± 1.101)	*t*=3.743	0.007
		Upper V1	Congruent	Upward > Downward	*	−1.532 (± 0.436)	*t*=‐3.511	0.014
			*	Downward	Fast > Slow	4.288 (± 1.171)	*t*=3.610	0.035
	**High > Low**	*	Congruent	Upward > Downward	*	−0.616 (± 0.263)	*t*=2.343	0.034
				Upward	Fast > Slow	2.701 (± 0.894)	*t*=3.020	0.033
				Downward		5.265 (± 0.894)	*t*=5.889	< 0.001
				*		3.983 (± 0.568)	*t*=7.008	< 0.001
		Upper V1		Upward > Downward	*	−1.238 (± 0.301)	*t*=‐4.116	0.003

^a^ Results were average over the respective level.

**FIGURE 5:**
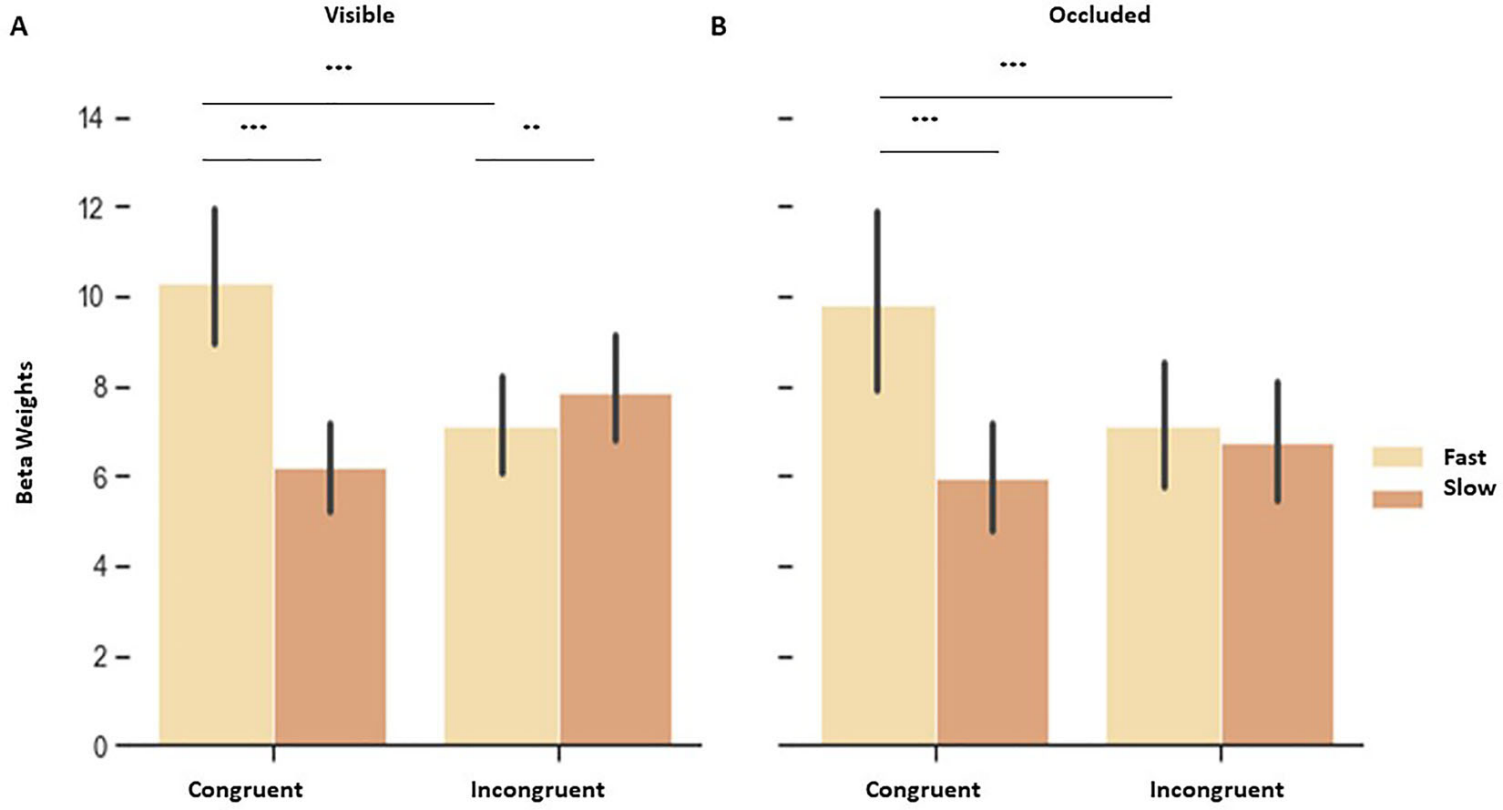
Beta weights of congruent and incongruent trials in visible and occlusion phases. Fast congruent trials elicited enhanced fMRI responses in V1 during **(A)** Visible and (B) Occluded phases, compared to slow congruent and incongruent conditions. Note, however, that slow incongruent (speeded‐up stimulus in the vertical trajectory) elicited higher responses compared to fast incongruent (slowed‐down stimulus in the vertical trajectory).

*Low predictable context*: During visible stimulation in the low predictable context, we observed again direction and V1 Quadrant interaction (Figure 3B—F (1, 14) = 26.231, *p* < 0.001, ŋ_p_^2^ = 0.652, see Table 2 for post‐hoc analyses), which demonstrated that the insertion of the noise through the incongruent trials still led to higher responses in the opposite quadrant; this effect was significant for upper V1 and marginally significant for lower V1. Indeed, in the congruent condition, fast motion elicited higher responses in V1 than slow motion, whereas the incongruent trials with changes in velocity led to intermediate responses compared to fast and slow congruent trials. Statistically, an interaction between velocity and congruency (Figure 5A) was found with higher fMRI signals during congruent fast motion and incongruent slow motion (F (1, 14) = 99.931, *p* < 0.001, ŋ_p_^2^ = 0.877).

We further observed an interaction between direction and velocity (F (1, 14) = 4.694, *p* = 0.048, ŋ_p_^2^ = 0.251) and a marginally significant triple interaction between direction, velocity, and V1 Quadrant (Figure 4B), which yielded significant post‐hoc comparisons (F (1, 14) = 4.240, *p* = 0.059, ŋ_p_^2^ = 0.232).

*High × Low predictability*: When comparing beta weights of congruent trials from both contexts, we observe interactions between direction and V1 Quadrant (F (1, 14) = 54.439, *p* < 0.001, ŋ_p_^2^ = 0.795; see Table 2 for all post‐hoc analyses), which indicated that the vertical stimulations were salient enough to elicit higher responses in the opposite quadrant. Additionally, triple interactions were seen between direction, velocity and V1 Quadrant, again highlighting that velocity played a role in V1 responses (F (1, 14) = 12.864, *p* = 0.003, ŋ_p_^2^ = 0.479) and velocity, V1 Quadrant, and predictability (F (1, 14) = 6.408, *p* = 0.024, ŋ_p_^2^ = 0.314; though no significant effects were found in the post‐hoc comparisons between different contexts). Finally, a main effect of velocity was observed (F (1, 14) = 78.946, *p* < 0.001, ŋ_p_^2^ = 0.849).

#### 4.2.2 Occluded Phase

*High predictable context*: Beta weights of the high predictable occluded phase indicated an interaction between direction and V1 Quadrant (Figure 3C), indicating that during occlusion, downward direction elicited a similar pattern of responses in upper V1, but not lower V1, compared to the visible phase (F (1, 14) = 10.927, *p* = 0.005, ŋ_p_^2^ = 0.438 (see Table 2 for post‐hoc analyses). Further, a main effect of velocity was found (F (1, 14) = 42.861, *p* < 0.001, ŋ_p_^2^ = 0.754). Since we observed the triple interaction between direction, velocity, and VF in the visible phase (Figure 4C), we also computed post‐hoc for the occluded phase and found significant differences between upward fast versus upward slow in lower V1; downward fast versus downward slow in lower V1; upward fast versus upward slow in upper V1; and downward fast versus downward slow in upper V1.

*Low predictable context*: During the low predictable context, we once more observed an interaction between direction and V1 quadrant (Figure 3D–F (1, 14) = 19.683, *p* < 0.001, ŋ_p_^2^ = 0.584; post‐hoc comparisons revealed the difference only when incongruent trials were not included in the analysis: F (1, 14) = 14.691, *p* = 0.002, ŋ_p_^2^ = 0.517. Additionally, interactions between velocity and congruency were found (Figure 5B–F (1, 14) = 30.281, *p* < 0.001, ŋ_p_^2^ = 0.684, as well as direction and congruency (F (1, 14) = 15.575, *p* = 0.001, ŋ_p_^2^ = 0.527). Further, results revealed a marginally significant interaction between direction and velocity (F (1, 14) = 4.016, p = 0.065, ŋ_p_^2^ = .223). For the sake of completeness, we computed post‐hoc analyses on the triple interaction between direction, velocity, and VF (Figure 4D) and observed a significant difference between downward fast versus downward slow in upper V1.

*High x Low predictability*: During occlusion, the comparison between both contexts yielded significant interactions of direction and V1 quadrant (F (1, 14) = 18.134, *p* < 0.001, ŋ_p_^2^ = 0.564, see Table 2), and a marginally significant interaction of direction and velocity (F (1, 14) = 3.451, *p* = 0.084, ŋ_p_^2^ = 0.198). We further observed a main effect of direction (F (1, 14) = 5.489, *p* = 0.034, ŋ_p_^2^ = 0.282) and velocity (F (1, 14) = 49.106, *p* < 0.001, ŋ_p_^2^ = 0.778), but no main effect of or interaction with predictability.

### 4.3 Trial‐by‐Trial Information in LP Context

**FIGURE 6:**
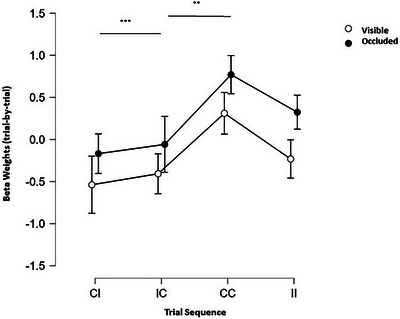
Trial‐by‐trial comparison for visible and occluded phases. The trial sequence abbreviations are CC = congruent trials after congruent trials, CI = congruent trials after incongruent trials, IC = incongruent trials after congruent trials, CC = congruent trials after congruent trials, and II = incongruent trials after incongruent trials.

The results above showed that there are differences between congruent and incongruent trials in both visible and occluded conditions. However, differences in BOLD response might not only be due to the cumulative trial history (i.e., LP vs. HP context) but also to the recent trial history. To this end, we analyzed the trial‐by‐trial fMRI responses in V1 to test whether responses to congruent trials after incongruent trials (and vice versa) are higher due to lower predictability (i.e., congruent followed by congruent or incongruent followed by incongruent trials—Figure 6). We performed a repeated measures ANOVA (2×2×4: visible/occluded; V1up/V1down; congruence: congruent trials followed by incongruent trials [CI], incongruent trials followed by congruent trials [IC], congruent trials followed by congruent trials [CC], incongruent trials followed by incongruent trials [II]). We found significant effects only for congruence (F (3, 45) = 4.678, *p* = 0.006, ŋ_p_^2^ = 0.059; CI vs. CC: MD = ‐0.894, ± 0.260, *t* = ‐3.441, p_bonf_ = .008; IC vs. CC: MD = ‐0.774, ±.260, t = ‐2.978, *p*_bonf_ = 0.028), and no difference between visible and occluded phases (F (1, 15) = 1.643, *p* = 0.219, ŋ_p_^2^ = 0.023).

### 4.4 Multivariate Pattern Analysis Results

To test whether the informational content in V1 was similar during visible and occluded conditions, we performed trial‐wise searchlight MVPA analyses in HP and LP contexts separately. Additionally, a comparison between both contexts was carried out to directly compare the informational content in both contexts. A multivariate analysis of trial‐history effects was not possible due to the low number of trials in some conditions. Tables 3 and 4 depict the accuracy values of all analyses, of which we decoded spatial information, i.e., upward versus downward trajectory, and temporal information, i.e., fast versus slow motion, respectively.

**TABLE 3:** Decoding accuracy of temporal information: Accuracy values of all analyses of all conditions are displayed on the table above, together with the standard error of the mean (SE). Permutation p‐values demonstrate that the permuted accuracy distributions were significantly lower than the true label accuracy distribution. The average number of spheres included in the sample of 5% most informative spheres is in the range of 81 to 132.

Temporal information: Velocity							
ROI		Predictability	Accuracy	SE	Permutation *p*‐value	N spheres (average)	Paired *T*‐Test
**Lower V1**	**Visible‐Occluded**	High	0.577	0.005	< 0.001	132 (± 52.52)	*t* = 3.487. *p* = 0.003
		Low	0.561	0.004	< 0.001	125 (± 50.57)	
**Upper V1**		High	0.577	0.005	< 0.001	100 (± 63.11)	*t* = 3.208. *p* = 0.006
		Low	0.562	0.004	< 0.001	95 (± 61.80)	
**V5**		High	0.569	0.005	< 0.001	81 (± 18.38)	*t* = 3.390. *p* = 0.004
		Low	0.558	0.004	< 0.001	73 (± 20.93)	
**ROI**		**Predictability**	**Accuracy**	**SE**	**Permutation *p*‐value**	**N spheres (average)**	**Paired *T*‐Test**
**Lower V1**	**Visible**	High	0.586	0.006	< 0.001	112 (± 44.48)	*t* = 1.352. *p* = 0.196
		Low	0.576	0.004	< 0.001	94 (± 42.10)	
**Upper V1**		High	0.581	0.007	< 0.001	86 (± 51.98)	*t* = ‐0.192 *p* = 0.850
		Low	0.582	0.005	< 0.001	75 (± 46.26)	
**V5**		High	0.580	0.005	< 0.001	73 (± 20.16)	*t* = 0.484 *p* = 0.636
		Low	0.577	0.005	< 0.001	58 (± 20.87)	
							
**ROI**		**Predictability**	**Accuracy**	**SE**	**Permutation *p*‐value**	**N spheres (average)**	**Paired *T*‐Test**
**Lower V1**	**Occluded**	High	0.573	0.003	< 0.001	124 (± 47.70)	*t* = 1.120 *p* = 0.280
		Low	0.568	0.005	< 0.001	107 (± 45.36)	
**Upper V1**		High	0.576	0.004	< 0.001	94 (± 60.94)	*t* = 1.054 *p* = 0.308
		Low	0.571	0.004	< 0.001	86 (± 59.31)	
**V5**		High	0.581	0.005	< 0.001	76 (± 18.42)	*t* = 1.359 *p* = 0.194
		Low	0.575	0.004	< 0.001	67 (± 18.63)	

**TABLE 4:** Decoding accuracy of spatial information: Accuracy values of all analyses of all conditions are displayed on the table above, together with the standard error of the mean (SE). Permutation *p*‐values demonstrate that the permuted accuracy distributions were highly significantly different from the true label accuracy distribution. The average number of spheres in the sample, which consists of 5 % most informative spheres, ranged from 45 to 112.

Spatial information: Direction							
ROI		Predictability	Accuracy	SE	Permutation *p*‐value	N spheres (average)	Paired *T*‐Test
**Lower V1**	**Visible‐Occluded**	High	0.551	0.005	< 0.001	100 (± 42.93)	*t* = ‐2.501 *p* = 0.024
		Low	0.564	0.005	< 0.001	96 (± 42.84)	
**Upper V1**		High	0.545	0.004	< 0.001	71 (± 46.59)	*t* = ‐2.579. *p* = 0.021
		Low	0.554	0.005	< 0.001	77 (± 48.21)	
**V5**		High	0.548	0.004	< 0.001	66 (± 21.61)	*t* = ‐5.628 *p* < 0.001
		Low	0.571	0.005	< 0.001	72 (± 23.80)	
							
**ROI**		**Predictability**	**Accuracy**	**SE**	**Permutation *p*‐value**	**N spheres (average)**	**Paired *T*‐Test**
**Lower V1**	**Visible**	High	0.594	0.005	< 0.001	101 (± 44.52)	*t* = ‐4.303 *p* < 0.001
		Low	0.629	0.010	< 0.001	112 (± 48.75)	
**Upper V1**		High	0.585	0.009	< 0.001	73 (± 57.19)	*t* = ‐3.373. *p* = 0.004
		Low	0.612	0.011	< 0.001	83 (± 56.88)	
**V5**		High	0.553	0.003	< 0.001	49 (± 24.03)	*t* = ‐5.378. *p* < 0.001
		Low	0.581	0.005	< 0.001	59 (± 20.77)	
							
**ROI**		**Predictability**	**Accuracy**	**SE**	**Permutation *p*‐value**	**N spheres (average)**	**Paired *T*‐Test**
**Lower V1**	**Occluded**	High	0.554	0.005	< 0.001	74 (± 39.94)	*t* = ‐4.763. *p* < 0.001
		Low	0.571	0.004	< 0.001	88 (± 38.88)	
**Upper V1**		High	0.556	0.005	< 0.001	45 (± 33.92)	*t* = ‐2.548. *p* = 0.023
		Low	0.569	0.006	< 0.001	62 (± 43.10)	
**V5**		High	0.558	0.004	< 0.001	51 (± 23.52)	*t* = ‐3.740. *p* = 0.002
		Low	0.578	0.005	< 0.001	58 (± 21.17)	

Endnotes.

#### 4.4.1 Temporal Information: Classifying Velocity

*Classifying velocity patterns of occluded phase from visual phase in HP and LP contexts*. Here, we trained the classifier in the visible phase and tested in the occluded phase data using fast and slow conditions as labels, separately for HP and LP contexts. Note that trajectory changes up/down were collapsed, so only velocity can possibly explain any results. We focused our analysis on the primary visual areas (see Supplementary Material for additional results in area V5/human MT+). Decoding accuracies, obtained from the sample of the 5% most informative spheres (range of 81 to 132 spheres), were above chance level (Table 3: Visible‐Occluded), suggesting that temporal information is represented in visual areas. Importantly, the representational patterns of temporal information are similar during visible and occluded phases.

#### 4.4.2 Spatial Information: Classifying Direction

*Classifying direction patterns of visible phase from occluded phase in HP and LP contexts*. In these analyses we trained the classifier in the visible phase and tested in the occluded phase data using upward and downward conditions as labels, separately for HP and LP contexts. Note that the results from both sessions with opposing velocity association were collapsed so that a decoding of stimulus velocity cannot explain the results below. Here we expected to decode information from the occluded data with training in the visible phase, as previously observed (Agostino et al. 2023). In order to obtain the most informative voxels, we averaged the 5% most informative spheres (range of 45 to 112 selected spheres). Accuracies above chance level were found, suggesting that we could successfully decode direction‐specific informational patterns from the visible phase in the occluded phase in the lower and upper V1 and V5. Additionally, all analyses with the true labels yielded significantly higher accuracies compared to the permutation analyses (Table 4: Visible‐Occluded). Together, these findings indicate that MVPA significantly extended the results from the univariate analyses by showing that both phases share a similar informational pattern in the primary visual area. Furthermore, these classification analyses replicate the results of our previous study, in which we demonstrated a common engagement of low‐level visual cortex during the presentation of visible and dynamically occluded motion.

*Manipulation checks*: As a manipulation check for spatial decoding, we tested the classifier in visible and occlusion phases separately (Table 4: Visible; Occluded). Results indicated that accuracies from the classification analysis in which we trained and tested in visible phase data were higher compared to the two other classification analyses. Such results were expected, as during the visible period more visual information is available to be encoded by the visual system. In contrast, the other two analyses presented very similar averaged decoding accuracies. Also note that the decoding accuracies of this analysis were generally smaller compared to a previous study (Agostino et al. 2023) as we used trial‐wise rather than run‐wise estimates as input to the MVPA, which includes an increased noise level, while the previous study used run‐wise averages.

As a manipulation check for the temporal decoding, we analyzed visible and occluded phases separately. Note, however, that, in comparison with the decoding of spatial information, we did not observe higher accuracy for visible phase analysis compared to the other two analyses (Table 3: Visible; Occluded). This may indicate that the pattern of temporal information was encoded similarly, independent of the availability of the stimulus (visible or occluded). In contrast with the comparison of spatial information decoding accuracies from HP and LP contexts, in which we observed higher accuracies for the latter, accuracies from analyses with LP context were not higher than accuracies from analyses with HP context data (Table 4). Instead, classification analyses with visible phase only or occluded phase only did not yield significant different results between both contexts, whereas analyses in which the classifier was trained in visible and tested in occluded HP context data yielded higher accuracies than the same analysis with LP context data. These findings might suggest that temporal information can be better decoded when the encoding is not affected by some noise, such as the reliability of the stimulus velocity.

When comparing accuracies of HP and LP context, we observed significantly higher accuracies decoded from LP context data compared to accuracy decoded from HP context data in all analyses; this may suggest that the incongruent condition might have led to an encoding of more information. Therefore, we directly compared congruent and incongruent decoding.

#### 4.4.3 Congruent Information in LP Context

**FIGURE 7:**
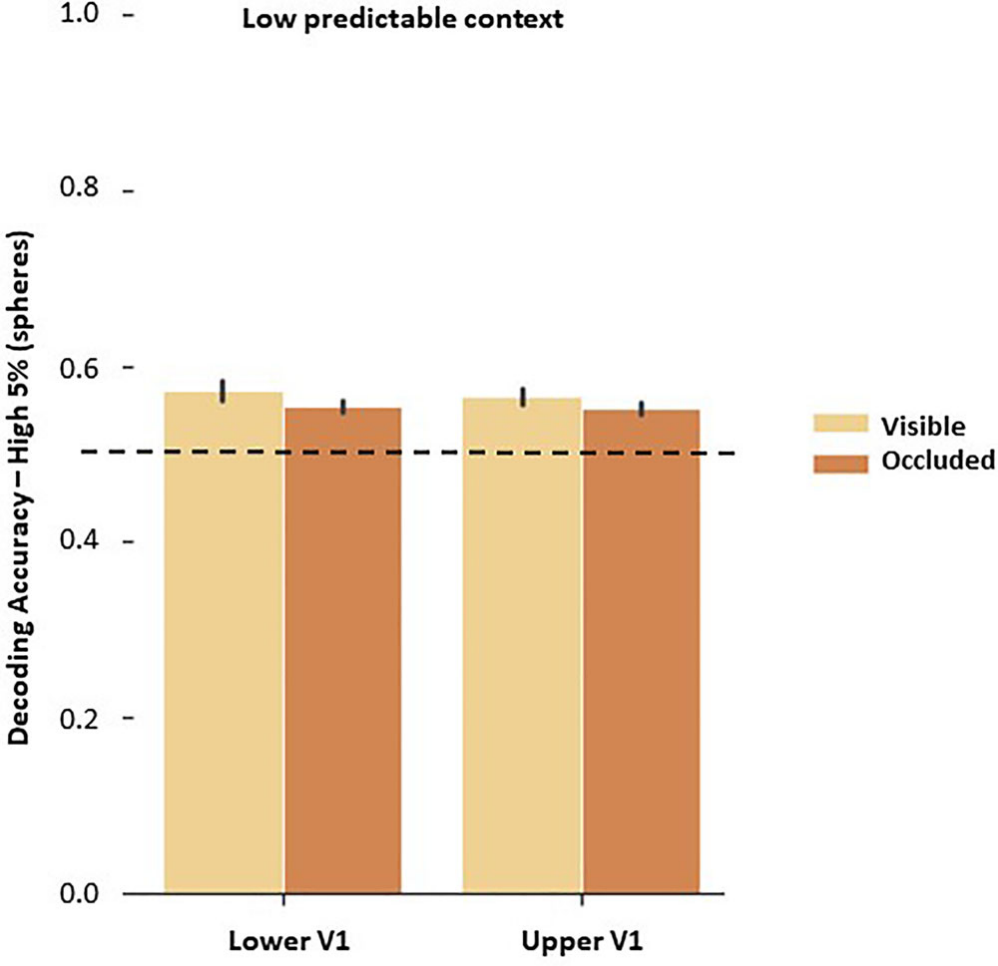
Classifying congruent and incongruent information. Yellow bars depict the averaged accuracy of classification analyses in which the classifier was trained and tested in the visible phase (“Visible”), and orange bars depict the averaged accuracy of classification analyses in which the classifier was trained and tested in the occluded phase (“Occluded”). The dashed line on 0.5 represents the theoretical chance level.

*Classifying incongruent trials of visible phase and occluded phase in LP context*: These analyses were carried out by training the classifier in the visible and occluded data separately, using congruent and incongruent conditions as labels. Results (Figure 7) indicated that averaged accuracies (average of the number of the 5% most informative spheres is 75, ± 41.05) were significantly above chance level compared to permutation analyses of visible phase in lower V1 (mean (M) = 0.572, ± 0.024, permutation *p* (*p*_perm_) < 0.001; upper V1 (M = 0.566, ± 0.019, *p*_perm_< 0.001) and V5 (M = 0.571, ± 0.022, *p*_perm_< 0.001), as well as of occluded phase in lower V1 (M = 0.554, ± 0.015, *p*_perm_< 0.001), upper V1 (M = 0.552, ± 0.014, *p*_perm_< 0.001) and V5 (M = 0.580, ± 0.023, *p*_perm_< 0.001). These results indicated that incongruent trials were successfully classified to be different from congruent trials, suggesting that different information was encoded during the tasks with lower temporal predictability. While this difference might be due to the observable changes in speed during the visible phase, this argument does not account for the differences found during the occluded phase. Finally, these findings significantly extend the univariate analysis, which showed different patterns of activity between congruent and incongruent conditions.

#### 4.4.4 Overlap of Sphere Centers

In our previous study (Agostino et al. 2023), we used the 5% highest accuracy approach and showed that the overlap of significant spheres across analyses is above 80% and at the locations of V1, which represented the stimulus trajectory.

To evaluate that our approach of choosing the 5% highest accuracy values is robust and free of bias and does not randomly select different voxels within a region for the different analyses in the current study, we again calculated the number of spheres that share the same centers across different classification analyses. Interindividual results indicated that all participants shared spheres across different analyses in different probability contexts in all masks. The averaged overlapped sphere center for all classification analyses performed here can be seen in Tables 1 and 2. The overall average of all analyses is 83.19 (SD: ± 21.63). Note that this value is similar to the number of sphere centers reported in the previous study (Agostino et al. 2023). This similarity between overall averages of both studies may be a result of the down‐sampled space, as the analyses were carried out in the reduced space of ROI masks. Together, these results suggested that our approach of selecting the 5% highest informative voxels is robust, supporting the interpretation that when different decoding analyses share the same sphere center, the decoded pattern is truly informative, rather than a random selection of noise.

## 5 Discussion

This study investigated whether temporal motion extrapolation mechanisms would be affected by high versus low predictable contexts and whether the behavioral effects are related to differential fMRI responses in the primary visual cortex during visible and occlusion periods. Our behavioral results demonstrated that during the visible phase, differences were observed between HP and LP contexts, indicating that performance was higher for HP compared with LP, especially for slow motion in downward trajectory.

### 5.1 Behavioral Read‐outs

Reaction time results during visible stimulation showed that participants attempted to estimate the time‐to‐contact of the stimulus accordingly (faster responses during fast motion, slower responses during slow motion) in HP and LP contexts, following the expected pattern, and corroborating the results from our first study (Agostino et al. 2023). However, we further observed an interaction between velocity, predictability, and direction, which indicated that participants responded faster to slow downward motion during the LP context. During occlusion, temporal estimation in the LP context did no longer follow the same pattern, meaning that faster trials were not estimated faster and slower trials were not estimated longer. Rather, reaction times were time‐compressed across conditions in the LP context, suggesting that when accurate temporal estimation was impossible in 30% of the cases, participants used an averaging strategy. It could be speculated that this effect might be mediated by post‐error adaptations (Danielmeier and Ullsperger 2011; King et al. 2010). Post‐error adaptations alter future behavior, leading to potential improvements, such as faster reaction times or higher accuracies. One of these adaptations is known as post‐error speeding, and it has been shown that it is related to an enhancement of performance after a given threat (Caudek et al. 2015) and an increase of activity in task‐relevant visual areas (King et al. 2010). However here, we didn't observe the improvement of fast stimulus estimation, as participants were still trying to adequately respond according to the learned pattern. This was also found in a reversal of responses for incongruent relative to congruent trials during occlusion, pointed out by reaction time error, which suggests that participants applied the learned congruent direction‐velocity change (see Supplementary Material). Summing up, the pattern of behavioral temporal estimates indicates that participants performed well in both contexts in the visual phase with some overestimation of TTC in the fast condition in accord with previous publications. During occlusion, however, an increase in RT errors was observed for the LP context relative to the HP context.

### 5.2 Univariate fMRI Read‐outs

At the neural level, we observed V1‐quadrant‐specific responses to upward and downward motion, as expected. No significant differences were found between the HP and LP contexts, neither during the visible nor during the occlusion phase. This finding is not in line with our initial hypothesis of enhanced fMRI signals in the LP context. However, previous studies that observed an enhancement in activity in the primary visual cortex during the processing of dynamically occluded stimulation due to attention did not modulate attentional demands parametrically (Kok et al. 2012; Coull et al. 2008; Doherty et al. 2005). In particular, Coull et al. (2008) reported results apparently contradicting the postulates of the predictive coding model. Using a time‐to‐contact (TTC) paradigm, the authors investigated how fMRI responses in low‐level visual areas were modulated by temporal predictability during egocentric (subjective viewpoint) and allocentric (external viewpoint) viewing. In an ecologically valid driving simulation, participants were instructed to predict whether a car would touch a wall in one task and, in another task, whether the color of the car and the wall matched. It was hypothesized that proper attentional allocation would enhance stimulus detection, considering that spatiotemporal predictability is implicitly related to the object‐motion TTC task, while temporal predictability is explicitly associated with the temporal cueing task. The authors observed increased fMRI responses in V1 for TTC prediction during the egocentric judgments, likewise a variation in activity as a function of increasing certainty of collision, while allocentric judgments selectively enhanced responses in V5.

These findings were interpreted as an effect of attentional modulation of activity in V1. Moreover, this interpretation is in line with the findings of another electrophysiological study, which investigated how temporal and spatial information during dynamically occluded stimulation can expand attentional resources applied during the occlusion period (Doherty et al. 2005). The authors observed that the P1 component, which represents accumulated activity of many ventral and dorsal extrastriate visual regions (Di Russo et al. 2002; Foxe et al. 2005), was enhanced when spatial and temporal expectations were combined, suggesting that temporal predictability of an occluded target plays an important role in establishing the efficacy of sensory processing. Moreover, they observed that spatial and temporal predictability work synergistically together, and this interaction may affect the attention allocation to the reappearance of the occluded moving stimulus (Doherty et al. 2005).

Some researchers have tried to reconcile the opposing views generated by those studies that found evidence that predictability reduces response in the primary visual cortex with those that found the opposite pattern. For instance, Kok and colleagues (2012) tested two hypotheses related to this contraction: (1) Attention and prediction have an opposing relationship, meaning that the excitatory attentional effect outweighs the inhibitory effect related to prediction by enhancing activity when predicted attended stimuli are presented compared to unattended predicted stimulation; (2) attention and prediction have an interactive relationship, meaning that attention boosts the precision of prediction, resulting in an enhanced activity promoted by the predictive error. The authors tested these hypotheses by measuring fMRI responses in bilateral low‐level visual areas (attended and unattended sides) while presenting cues that indicated the likelihood of the side where a Gabor patch would appear. Participants were instructed to indicate the orientation of the Gabor patch on the attended side and to ignore Gabor patches on the unattended side. Results indicated that reduced responses in V1 were observed on the unattended side when they were expected there, compared to enhanced responses in V1, V2, and V3 on the attended side for expected stimuli. The study provided evidence supporting the first hypothesis, which suggested that attention cancels the response reduction during high predictability, as the excitatory response related to attention enhances activity in low‐level visual areas, but only on the attended side. Recently, we showed that when participants are asked to estimate the end positions of a partially occluded traveling stimulus, responses in V1 were enhanced along the occluded trajectory. Remarkably, even though the end position was always associated with a specific velocity, hence predictable, the participant's engagement during the occlusion phase increased activity in V1 (Agostino et al. 2023). Note that the trajectory was not straight but complex with a 90° turning point, which may have added an extra challenge to the visual processing and an extra strain on the attentional resources.

All in all, it is conceivable that attention acts in an all‐or‐none way and would therefore increase the signal to the maximum in both contexts. Hence, attention would affect prediction processing not gradually but categorically, as indicated by Fischer et al. (2013), who investigated the relation between temporal predictability and temporal attention and reported that V1 could be primed and more responsive to temporally predictable stimuli. Moreover, it is important to emphasize that during occlusion participants did not make a simple time‐to‐contact judgment, but rather they were required to make a more complex prediction due to the velocity‐direction association. Potentially, the reappearance of the stimulus may have automatically captured attention and thus may have generated a response high enough to decrease the pattern of activity related to the predictive error, thus equalizing the response pattern in HP and LP contexts. (See also below, where we discuss the results at the trial level, which may support predictive coding to some extent).

We further observed that upward trajectories enhanced responses in lower V1 and downward trajectories enhanced responses in upper V1, not only during the visible phase, but also, and most importantly, during the occlusion phase. These results corroborated our previous study (Agostino et al. 2023), which demonstrated that the mechanisms related to visualizing and extrapolating a moving stimulation indeed modulate activity in identical searchlights in low‐level visual areas. However, here we observed that the expected pattern was seen in the visible and occlusion phases in HP and LP contexts in upper V1, hence the lower visual field. Differences between vertical hemifields have been reported previously. The vertical meridian asymmetry (Carrasco et al. 2001; Rijsdijk et al. 1980; Previc 1990) indicates a dominance of the lower visual field over the upper visual field in different tasks, such as spatial resolution (Talgar and Carrasco 2002), visual acuity (Skrandies 1987), and motion (Levine and McAnany 2005), among others (Karim and Kojima 2010, for review). An ecological explanation for this difference between vertical meridians was given by Previc (1990), who proposed that the dominance of the lower visual field comes from the primordial nature of the primate visual system, which was functionally more developed due to forelimb manipulatory skills. Later, studies indicated that the lower VF contains a larger amount of “near‐preferring” neurons most commonly found in the latter compared to the first (Nasr and Tootell 2018, 2020; Karim and Kojima 2010). These findings could explain the difference between this and our previous study, given that in this study we introduced the reappearance of stimulus, which might also have led to an automatic capture of attention (Lakha and Humphreys 2005). It is important to mention once more that the portion (voxels) in the respective quadrants representing the position of the reappearance of the stimulus were carefully excluded from the analyses to avoid misleading conclusions.

While in the spatial domain the stimulation modulated different patterns of activity, in the temporal domain univariate results were unanimous. The presentation of fast stimulation consistently enhanced activity in both upper and lower visual quadrants. Interestingly, even during incongruent conditions of visible stimulation, there was a tendency for the incongruent slow motion (speeded‐up stimulus) to follow the same pattern of enhanced activity observed during the presentation of congruent fast motion. One possible explanation for these findings is that V1 may be more promptly receiving feedback projections from V5 during fast motion (Edwards et al. 2017; Sterzer et al. 2006; Muckli et al. 2005), which could increase the response signal, while processing of slow motion would be taking longer to reach V5 and reach V1 back. Note that the univariate results partially oppose the behavioral results in which we observed a major effect for slow rather than fast motion. However, during LP context, the reversal pattern seen during incongruent trials in reaction time could also be seen as a tendency in the univariate results.

Finally, our trial‐by‐trial analysis showed that beta values of trials following another trial of a different category (IC; CI) were higher than those of trials following trials of the same category (CC; II). These findings support our hypothesis that unpredictable stimuli enhance activity in V1, in accord with the notion of the predictive coding theory, suggesting that the averaging of congruent and incongruent trials ameliorated the different representational patterns of the conditions in the LP context. Moreover, the reappearance of the stimulus, which served as feedback, may have acted as a teaching signal, which indicates to the system that an error was committed or an expectation was violated. Such signals lead to behavioral adaptations, which could be observed in the participants’ potential strategy of averaging reaction time in order to optimize their temporal judgment (Ullsperger 2024).

It is worth mentioning that trial‐by‐trial GLM captures the temporal variability across trials, whereas the common GLM may average out some of these trial‐specific effects (Mumford et al. 2012). Moreover, by modeling each trial separately, the hemodynamic response variability particular to each trial and influenced by attention, arousal, and task engagement can be better captured compared to the common GLM, which may overlook or attenuate these trial‐specific effects (Mumford et al. 2012; Rissman et al. 2004; Dale and Buckner 1997). In sum, our univariate analyses revealed quadrant‐specific effects in V1 during visible stimulation and, to some extent, during occlusion. Moreover, differential fMRI responses were observed for the different velocities.

### 5.3 Multivariate Pattern Read‐outs

The series of multivariate pattern analyses extended the results of the univariate analyses. First, we observed that representational patterns of activity from the visible phase could be used to decode patterns from the occluded phase in upper and lower V1, thereby extending the results from the univariate analyses, where we only found effects in upper V1. Our results suggest that visible and extrapolated types of information share similar representational patterns in the very same voxels, replicating the results from our previous study (Agostino et al. 2023). The appropriateness of the sphere selection approach that we used was further confirmed by computing the overlap of sphere centers. This overlap indicated that the majority of spheres shared the same center in upper or lower V1 across different types of classification (train in visible, test in occluded; train and test in visible; and train and test in occluded), suggesting that the algorithm was decoding relevant information rather than noise, and also corroborating our previous study. Importantly, the effect was observed both in HP and LP contexts.

However, when comparing the accuracy values of HP with LP context, we observed significantly higher accuracy values in the LP context. These results may be due to enhanced attention in the LP context in which stimuli had to be monitored closely after they changed direction to detect a possible change in speed. Moreover, the change in velocity might have led to a difference in brain activity. Indeed, we observed a difference between congruent and incongruent trials when we classified congruent and incongruent trials from visible and occluded data separately, confirming that the representational patterns of congruent and incongruent trials were significantly different. The results from the visible phase indicate that a change in stimulus can be detected using MVPA. Importantly, the results from the occluded phase indicate that this effect is not due to the change in stimulus properties but due to the expectation violation, as no change in speed was observable there. This pattern of results also suggests that the observed effects in V1 might be at least partially due to feedback, as they depended on the reappearance of the stimulus. Importantly, this region of V1, which encoded the trajectory of the stimulus after reappearance, was not included in the analysis thus, any observed effects must be due to feedback mechanisms, for instance, from higher visual areas (e.g., V5 Alink et al. 2010; Noesselt et al. 2002), frontal areas (Summerfield et al. 2006), or even hippocampal regions (Ekman et al. 2023). Moreover, our findings are in line with a recent study in mice that also investigated expectation violation in V1 (Tang et al. 2023). The study revealed that when a stimulus violated the mice's expectation, the neural response of V1 was enhanced, leading to a better encoding of the sensory information. The effect could be seen in both firing rate and neuronal synchrony in V1.

Future fMRI studies with increased resolution could disentangle the effects of congruent and incongruent trials by segregating the areas of V1 that represent the horizontal and vertical trajectories in greater detail and would thus be able to separately test the similarity of responses for fast and slow velocities in congruent and incongruent trials. Moreover, the effect of attention in motion extrapolation needs to be tested by parametrically modulating attentional demands and comparing whether the effect remains unchanged.

In conclusion, we tested whether visible motion processing or motion prediction would differentially modulate fMRI responses in the primary visual cortex in higher or lower predictability contexts. According to the predictive coding model, it was expected that stimulation presented in low predictable contexts would enhance activity in V1 compared to high predictable contexts. However, alternatively, activity during a highly predictable context would also increase activity in this region if any amount of attention outweighs the predictive mechanism in an all‐or‐nothing rather than a gradual way. Our results provided evidence supporting the latter hypothesis by showing no difference in fMRI‐response signal of high‐and low‐predictable‐context tasks when analyzing the effects of accumulated evidence. When analyzing short‐term adaptation effects, our trial‐history analysis pointed to differences between consecutive trials of different categories compared to the same category, suggesting that volatility at the trial level may have increased activity in V1 and might thus be the more relevant time scale when analyzing effects in V1. This difference was further supported by our MVPA results, which showed a difference in the decoding of the representational pattern of congruent and incongruent trials.

## Author Contributions

**CSA**: conceptualization, software, formal analysis, investigation, writing–original draft, visualization. **HH**: resources, writing–review and editing, funding acquisition. **TN**: conceptualization, supervision, writing–original draft, funding acquisition.

## Ethics Statement

This study was approved by the ethics committee of the Otto‐von‐Guericke‐University.

## Conflicts of Interest

The authors declare no conflicts of interest.

## Peer Review

The peer review history for this article is available at https://publons.com/publon/10.1002/brb3.70769.

## Supporting information




**Supplementary Material**: brb370769‐sup‐0001‐SuppMat.docx

## Data Availability

Data will be shared upon request with the need for approval from the requesting researcher's local ethics committee.

## Endnotes

1 The first subjects had few volumes less due to the adjustment of scanning time and the end of the experiment. One participant had eight volumes less, and four participants had five volumes less at the end of each run.
